# Targeting the Cellular Signaling: BRAF Inhibition and Beyond for the Treatment of Metastatic Malignant Melanoma

**DOI:** 10.1155/2012/259170

**Published:** 2011-12-15

**Authors:** Felipe Ades, Otto Metzger-Filho

**Affiliations:** ^1^Department of Dermatology, Institut Gustave Roussy, 114 rue Edouard Vaillant, 948005 Villejuif, France; ^2^Department of Medical Oncology, Institut Jules Bordet, Université Libre de Bruxelles, Boulevard de Waterloo, 121, 1000 Brussels, Belgium

## Abstract

Although advances in cytotoxic treatments have been obtained in several neoplasias, in metastatic melanoma there was no drug able to significantly change the natural history of the disease in the last 30 years. In the last decade, translational research identified important mechanisms in malignant transformation, invasion, and progression. Signaling pathways can be abnormally activated by oncogenes. The identification of oncogenic mutated kinases implicated in this process provides an opportunity for new target therapies. The melanoma dependence on BRAF-mutated kinase allowed the development of inhibitors that produced major responses in clinical trials. This is the beginning of a novel class of drugs in metastatic melanoma; the identification of the transduction signaling networking and other “druggable” kinases is in active research. In this paper, we discuss the ongoing research on cellular signaling inhibition, resistance mechanisms, and strategies to overcome treatment failure.

## 1. Introduction

Malignant skin melanoma is one of the most chemoresistant and aggressive human neoplasias. In the last 30 years, no cytotoxic agent was able to importantly change the natural history of disease [1]. Several strategies to overcome resistance to cytotoxic agents have been tested, including combinations of drugs [2], cytokines, and vaccination [1]. With these therapeutic approaches, only a small fraction of the metastatic patients experienced tumour shrinkage, but such effects did not translate into significant clinical benefits in terms of progression-free survival or overall survival [1, 3]. Until recently, no predictive marker of response could be established.

This scenario started to change in the last decade. With advances in translational research, it was possible to identify pathways and somatic mutations implicated in the biology of the melanoma. The identification and blockade of abnormal signaling through the mitogen-activated protein kinase (MAPK) pathway is the most promising therapeutic strategy to date. Around 60% of all melanomas express somatic mutations in the BRAF protein, and 90% of these express the oncogenic activating V600E mutation [4]. Vemurafenib, an inhibitor of the V600E BRAF kinase activity, has produced major responses [5] and showed an overall survival advantage as single agent against dacarbazine in a recent phase III trial [6].

Despite the advances, responses are transitory and we have not yet been able to neither cure nor stabilize the disease for long periods. Better understanding of tumoral biology and its adaptations to the therapeutic intervention continues to be a challenge. Parallel signaling pathways and the network between them are some of the possible reasons for treatment resistance and could provide new targets to drug development. We reviewed the research advances in signaling pathway inhibition and new strategies to metastatic melanoma treatment.

## 2. MAPK Pathway—RAS/RAF/MEK/ERK

**Figure 1:**
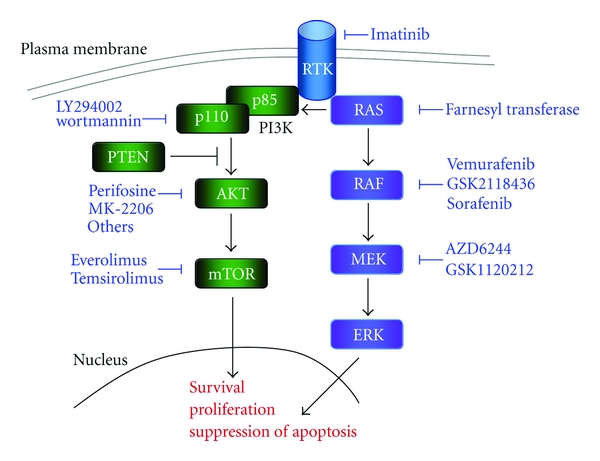
Schematic view of the MAPK and PI3K pathways. Drugs targeting the multiple kinases in the signaling cascades are represented in blue.

The MAPK pathway is frequently mutated in melanoma. It is involved in cell mutation, differentiation, and survival. In response to the extracellular signaling a RAS family protein recruits a RAF family protein to the cell membrane. Active RAS signals activate ERK and downstream effectors as shown in Figure 1. Specifically NRAS and BRAF mutations are highly associated with melanoma [4]. Preclinical results evidencing cytotoxic effects of the pathway blockage increased the clinical research interest in this direction [4, 7].

### 2.1. RAS

Oncogenic mutation in the sequence of the NRAS protein has been observed in around 15% of the melanomas [4] leading to protein activation and transduction of survival and proliferation signals [8, 9]. Preclinical research validated the RAS protein as a possible target for clinical drug development [7, 10].

Farnesyl transferase inhibitors are drugs developed to avoid membrane localization of RAS, preventing its activation [11]. These drugs have been evaluated across several neoplasias with disappointing results [12, 13]. In melanoma, there was only one phase II study where no response was observed among the 14 patients [14]. The study did not stratify patients by their NRAS mutation status, which could explain the absence of response in this cohort of patients. There is some evidence of synergic mechanisms of farnesyl transferase inhibitors with cisplatin [15], but this approach was not clinically tested.

### 2.2. RAF

Almost 60% of melanomas show a BRAF mutation [4, 16]. Among these patients, the most common is the replacement of the glutamic acid by the valine aminoacid in the position 600, the so-called V600E BRAF mutation [4]. It accounts for 90% of the mutations of the BRAF gene [4].

V600E BRAF is a frequent mutation in melanoma and is also commonly found in benign nevi [17]. BRAF mutations do not seem sufficient to promote malignant transformation, but may play a role in early stages of carcinogenesis [16, 18–20]. A second oncogenic hit could be necessary: the interaction of BRAF with PTEN [21], p16 [20], p53 [18], AKT [22], and UV radiation [23] has already been described.

The first therapeutic approach to block this protein was the use of multikinase inhibitor sorafenib, which targets BRAF, CRAF, PDGFR*β*, and VEGFR [24]. Sorafenib was used as monotherapy and combined with cytotoxic chemotherapy—dacarbazine [25], temozolomide [26], and carboplatin plus paclitaxel [27]—with negative results. That contrasts with positive results in renal cell carcinoma [28] and suggests that melanoma could be specifically more dependent of the BRAF pathway. Some authors argue that sorafenib is not an effective BRAF inhibitor in the doses used in the clinical trials [29].

Vemurafenib, also known as PLX4032, RG7204, or RO5185436, is potent and specific inhibitor of the V600E BRAF activity, recently approved for the treatment of advanced melanoma. In a phase I/II study, after the escalation dose phase, 32 melanoma patients harboring the V600E BRAF mutation were included in the twice daily 960 mg dose regimen to evaluate response rate. An impressive RR of 81% was observed, including 24 partial responses and two complete responses. Vemurafenib was active even in patients with multiple lines of treatment, high LDH levels, or visceral metastasis. The estimated progression-free survival was more than seven months, and at the time of the publication, the overall survival had not been reached [5]. Vemurafenib was tested in a phase III randomized trial against dacarbazine. Interim analysis for overall survival showed a survival advantage for vemurafenib with a relative risk reduction of death of 63% (95% confidence interval [CI], 0.26 to 0.55; *P* < 0.001). At 6 months, overall survival was 84% (95% CI, 78 to 89) versus 64% (95% CI, 56 to 73), the estimated median progression-free survival was 5.3 months versus 1.6 months, and response rate was 48% versus 5% in the vemurafenib versus the dacarbazine group [6]. Specific BRAF inhibition had the contradictory side effect of promoting proliferative skin lesions, arising from wild-type BRAF keratinocytes [30].

A second compound that targets the BRAF mutated protein is GSK2118436. It is an even more potent inhibitor of mutated BRAF kinase activity, with a less relevant cutaneous toxicity profile. One of the particularities of this compound is its ability to effectively pass the blood brain barrier. In a subgroup of patients enrolled in the phase I study, a reduction of the brain metastasis size was observed [31]. GSK 2118436 is under evaluation in a phase II study for the treatment of patients with brain metastasis [32]. In addition a phase III study is comparing GSK 2118436 to dacarbazine [33].

### 2.3. MEK

The MEK kinase is just downstream BRAF in the signaling pathway. Inhibition of this kinase is postulated as an interesting target in BRAF mutant melanoma [34], but not in NRAS [35]. In vitro studies identified higher sensibility of MEK inhibition in cells harboring the BRAF mutation [34]. Results from a phase I clinical trial showed disease response and stabilization in subgroups of melanoma patients [36] and the related translational research successfully identified the reduction of ERK phosphorylation [37, 38].

MEK inhibitors are under evaluation for the treatment of metastatic melanoma and have shown moderate activity in phase I studies [38]. The MEK inhibitor AZD6244 is being tested alone or in combination with chemotherapy in several phase II studies [39]. Importantly, not all the studies require the BRAF mutation as inclusion criteria. The molecule is also being tested in phase II studies both in combination with BRAF inhibitors [40] and after failure to BRAF inhibition [41].

### 2.4. ERK

Direct ERK inhibition is under investigation in basic research. An inhibitor of the kinase activity was synthesized and tested in mouse models of rheumatoid arthritis [42, 43]. Authors argue that this class of drug could be used in cancer treatment [43]. However, to date, there are no published data on ERK inhibition in this setting.

## 3. PI3K Pathway—PI3K/AKT/PTEN/mTOR

The PI3K pathway is activated by the biding of a ligand to a receptor tyrosine kinase (RTK) [44]. It interacts with multiple cellular mechanisms of survival, proliferation, mobility, differentiation, and growth [45]. Alterations in the signaling pathway can play a role in malignant transformation and invasion [45, 46]. Recently, it was demonstrated that the interaction of AKT with the mutated BRAF protein collaborates with melanoma development [47]. Several molecules targeting the signal transduction through this pathway are currently under development (Figure 1).

### 3.1. PI3K

Many anticancer therapies rely on apoptosis, and it has been postulated that the inhibition of PI3K activity could alter the process. LY294002 was developed as an inhibitor of PI3K activity. In melanoma cell lines, it effectively induced apoptosis both by itself and in combination with other drugs [48]. In mice models, the topical use of LY294002 in combination with a RAS inhibitor inhibited the melanoma graft invasive behavior and reduced angiogenesis [49]. The combination was also effective in cell lines [50].

The observation of the strong coexpression of the p110 fraction of the PI3K with the activation of mTOR motivated the strategy of double blocking those kinases [51]. Several combinations were tested in preclinical studies [51, 52].

PI3K and mTOR kinases belong to the phosphatidylinositol-3-kinase-related kinase (PIKK) family and share considerable homology in their active site. Inhibitors developed to block PI3K activity, like LY294002 and wortmannin, are, therefore, active against both kinases. It is likely that some of the effects of these compounds could be due not only to PI3K inhibition but to double blockage of the pathway. Research is being carried out in order for the small differences in the active sites to be understood and for specific inhibitors to be produced [53]. Clinical early-stage studies with double blocking drugs are recruiting.

### 3.2. AKT

Copy gains of the AKT3 gene are found in about 60% of melanomas and are correlated with melanoma progression [54]. The activation of the AKT pathway can suppress apoptosis [55].

Perifosine is the first compound inhibiting AKT to reach phase 2 studies in melanoma. It was administered to 18 melanoma patients and resulted in 7 disease stabilizations and 11 progressions after two 28-day cycles of treatment. Authors concluded that this drug should not be tested as a single agent [56]. Other compounds like MK-2206 [57, 58], RX-0201, PBI-05204, and GSK2141795 [59] are in early clinical development for several types of cancer [60–63].

It has been observed, in preclinical models, a cytotoxic synergistic effect of the combination of MK-2206 with other target therapies and conventional chemotherapy [58]. This approach could be particularly promising in malignant melanomas harboring the BRAF mutation, taking into account the role played by the interaction of those kinases in the malignant transformation.

### 3.3. PTEN

The phosphatase and tensin homolog (PTEN) is a tumor suppressor gene [64, 65]. Its protein product inhibits melanoma growth and increases its susceptibility to apoptosis [66]. The deletion or silencing of PTEN increases the level of AKT3 phosphorylation in melanocytes and early stage melanoma cells [67, 68]. Cells lacking PTEN are more resistant to chemotherapeutic agents and show increased Bcl-2 activity, being more resistant to apoptosis [69].

In melanoma xenograft models in nude mice, the introduction of PTEN using a plasmid or chromosomal transfer inhibited tumor development [66]. It was also observed in breast cancer cell lines resistant to EGFR inhibition and lacking of PTEN activity that the introduction of the wild-type gene reverted the resistant phenotype [70]. Approaches restoring PTEN activity are still being under research at the basic laboratory setting.

### 3.4. mTOR

The mammalian target of rapamycin (mTOR) lays downstream AKT and can regulate its activity by feedback mechanisms. mTOR forms at least 2 active complexes of proteins: mTOR1 and mTOR2, the first of which suppress and the second activates AKT signalling [71].

mTOR signalling seems to be active in melanoma cell lines [72]. In breast cancer, inhibition of mTOR can reverse the trastuzumab resistance phenotype [73]. As a single-agent treatment, inhibition mTOR shows low activity and no clinical benefit against metastatic melanoma [74]. mTOR blocking is being tested in preclinical research in combination with heat shock protein vaccines [75] and MAPK inhibitors [71, 76].

Several derivatives of rapamycin targeting the mTOR1 complex are available and in clinical research. A number of phase I and -II studies testing the combination of everolimus and temsirolimus with conventional cytotoxic therapies are currently being carried out [77].

## 4. C-Kit Receptor

C-Kit belongs to the family of growth factors receptors. It is an RTK related to the process of melanocyte cell migration in embryogenesis [78]. It also plays a role in hematopoietic and germ cell homeostasis [79, 80]. Pathogenic activation of kit is observed in a number of solid tumors, such as seminoma [81], GIST [82], and thymic carcinoma [83].

A subset of melanomas (2 to 5%) also presents an amplification or mutation in c-Kit. It is more frequent in non-sun-exposed areas, such as in the mucous and acral melanomas subtypes, although it can be found in chronically sun-damaged skin areas [84, 85]. Most Kit mutations in melanomas are in the juxtamembrane region [86], which predicts responsiveness to imatinib mesylate, an inhibitor of tyrosine kinase activity [87].

Several case reports evidenced objective response of c-kit mutated melanoma to the use of imatinib [88–91]. This observation was also confirmed in cell-line studies [92]. When tested in phase II studies, discordant and disappointing negative results were obtained in three different trials [93–95]. The possible explanation to this finding is the relatively low dependence of the survival stimuli of c-Kit signaling or the need for a more specific and potent c-kit blocker. Different c-kit blockers are now in clinical development, and results from these trials may help answer these questions [96].

## 5. Resistance Mechanisms to BRAF Inhibition

**Figure 2:**
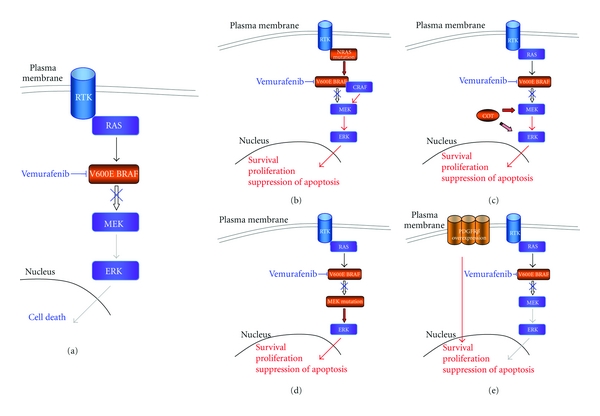
Representation of the resistance mechanisms to the vemurafenib. (a) Vemurafenib causes cell death by inhibiting V600E BRAF kinase activity and signaling transduction through the MAPK pathway. (b) New mutations in the NRAS causes heterodimerization of BRAF/CRAF and reactivation of the signaling pathway. (c) and (d) Phosphorylation of ERK independent of RAF stimulation is the result of the overexpression of COT (c) and of the additional mutation in MEK kinase (d). (e) Overexpression of PDGFR*β* can lead to resistance to vemurafenib treatment independent from the continuous inhibition of the MAPK pathway.

Inhibition of the mutated BRAF protein is the target therapy in more advanced clinical development. Even if most of patients responded to initial therapy, all of them would eventually relapse. The mechanisms related to resistance to BRAF inhibition are under intensive research. Reactivation of the MAPK pathway seems to be involved in the majority of the cases but signaling transduction by parallel pathway was also identified [97–99] (Figure 2).

The RAS protein is upstream from RAF. Activation of the MAPK pathway occurs as a result of the dimerization of RAF proteins (ARAF, BRAF, and CRAF) by the RAS stimuli. BRAF wild-type cells are resistant to BRAF inhibition because the biding of the drug to one dimmer causes the activation of the other [100–102]. In BRAF-mutated cells, the high activity of this kinase possibly causes a negative feedback in the RAS kinase [101], so the RAF dimerization in this population of cells is low, and the treatment is effective. In primary melanoma, mutations in RAS and RAF are mutually exclusive [4], but after vemurafenib treatment failure, it was identified a new mutation in the NRAS kinase, causing the reactivation of the MAPK pathway and disease progression [99] (Figure 2(b)). Experiments in cell lines overexpressing CRAF also showed resistance to vemurafenib effects [97].

Resistance can also rise from the activation of ERK independent of RAF signaling. The MAPK agonist gene *MAPK8 *codes for the protein COT. Overexpression of COT was detected in a subset of patients after failure to BRAF inhibition. This kinase can phosphorylate MEK and ERK in a RAF-independent way and mediate resistance to vemurafenib [97] (Figure 2(c)). Another resistance mechanism by the reactivation of the MAPK pathway is the acquisition or *de novo *activating mutation in the MEK protein [98] (Figure 2(d)).

PDGFR*β* overexpression was observed in a subset of cell lines resistant to vemurafenib. These cells were resistant to the antiproliferative effects of BRAF V600E inhibition despite sustained low ERK phosphorylation levels (Figure 2(e)). This observation was validated in a subgroup of patients [99].

New mutations in the BRAF protein confer resistance to its inhibition in cell lines [103], but to date it could not be identified in patients' biopsies.

## 6. Overcoming Resistance

As reactivation of the MAPK pathway seems to be involved in most of acquired resistance cases from BRAF inhibition [97–99], downstream blocking seems like a promising strategy. There are now several studies testing MEK inhibition in metastatic upfront and after anti-BRAF treatment failure. Concomitant or sequential inhibition of RAF and MEK may also be useful for such patients. Another strategy could be the development of double inhibition of BRAF and CRAF. Resistance arising from activation of COT or PDGFR*β* could be targeted by the combination of inhibitors of these proteins with maintenance RAF inhibitors.

Due to the variety of the resistance mechanisms, there is not a single strategy that will fit all patients. In the context of personalized therapy, tumor profiling is of major importance in different moments of the disease course. Only by the sequential analysis of the tumor molecular profile, can one identify the best target at the best moment for each specific patient.

Progress in melanoma treatment provides researchers with a unique opportunity to development novel therapeutic strategies. Laboratory resistance cell models coupled with the accessibility of melanoma skin tumours to sequential biopsies provides a research strategy that will lead to a better understanding of drug resistance mechanisms and improve clinical care.

## 7. Conclusion

Understanding the biology of the melanoma has been crucial for the development of new therapies. The observation of the dependence of the MAPK pathway for tumor survival boosted the research of methods for interfering with tumor cell signalization. Several compounds blocking multiple levels of the signaling pathways are being actively researched. The V600E BRAF inhibitor, vemurafenib, is now an approved agent for the treatment of advanced melanoma.

However, these advances are only available to around 60% of the patients, that is, those who have the BRAF mutation, and even in these patients the responses are transitory. For those not presenting this mutation, finding another target is urgent. Approaches like immunotherapy and vaccination are under development with promising results, but, again, only a small fraction of the patients respond to treatment (~10%) [104], and there are no available predictive markers of response.

Sequential biopsy and molecular profiling are important tools in cancer care and research. It allows us to understand the disease progression and its resistance mechanisms and to choose the most appropriate treatment strategy. Personalized molecular therapy is already a reality in malignant skin melanoma. The combination of kinase inhibition with conventional cytotoxic chemotherapy, with immunotherapy or multiple kinase inhibitions guided by the tumor molecular profile will provide new strategies for personalized melanoma treatment.
